# Overexpression of a NF-YC Gene Results in Enhanced Drought and Salt Tolerance in Transgenic Seashore Paspalum

**DOI:** 10.3389/fpls.2018.01355

**Published:** 2018-09-21

**Authors:** Xueli Wu, Haifan Shi, Zhenfei Guo

**Affiliations:** College of Grassland Science, Nanjing Agricultural University Nanjing, China

**Keywords:** drought, *NF-YC*, salinity, seashore paspalum, transformation, turfgrass

## Abstract

Seashore paspalum (*Paspalum vaginatum* O. Swartz) is an important warm-season turfgrass species. In this study we generated transgenic seashore paspalum overexpressing *CdtNF-YC1*, a nuclear factor Y transcription factor from hybrid bermudagrass (*Cynodon dactylon* × *Cynodon transvaalensis*). DNA blot hybridization and qRT-PCR analysis showed that *CdtNF-YC1* was integrated into the genomes of transgenic seashore paspalum plants and expressed. Reduced relative water content (RWC) and survival rate and increased ion leakage were observed in both wild type (WT) and transgenic plants after drought stress, while transgenic plants had higher levels of RWC and survival rate and lower ion leakage than the WT. Maximal photochemical efficiency of photosystem II (*F*_v_/*F*_m_), chlorophyll concentration and survival rate were decreased after salt stress, while higher levels were maintained in transgenic plants than in WT. In addition, an increased Na^+^ content and decreased or unaltered K^+^ in leaves and roots were observed after salt treatment, while lower level of Na^+^ and higher levels of K^+^ and K^+^/ Na^+^ ratio were maintained in transgenic plants than in WT. The results indicated that overexpressing *CdtNF-YC1* resulted in enhanced drought and salt tolerance in transgenic plants. Transcript levels of stress responsive genes including *PvLEA3, PvP5CS1*, *PvABI2*, and *PvDREB1B* were induced in response to drought and salt stress, and higher levels were observed in transgenic seashore paspalum than in WT. The results suggest that the enhanced drought and salt tolerance in transgenic seashore paspalum is associated with induction of a series of stress responsive genes as a result of overexpression of *CdtNF-YC1*.

## Introduction

Plant growth and development are often influenced by abiotic stress such as drought and salinity. Transcription factors are key players in plant adaptation to abiotic stresses by regulating downstream gene expression (Singh et al., 2002). Nuclear factor Y (NF-Y) transcription factor is consisted of three subunits namely NF-YA, NF-YB, and NF-YC. It regulates transcription of downstream genes by binding to CCAAT elements in approximately 25% of eukaryotic gene promoters (Nardini et al., 2013). NF-Y subunits are encoded by multigene families in plants,(Petroni et al., 2012), for example, there are 30 NF-Y members in *Arabidopsis* (Siefers et al., 2009) and 36 NF-Y members (seven *NF-YAs*, 17 *NF-YBs*, and 12 *NF-YCs*) in the model grass species *Brachypodium distachyon* (Cao et al., 2011). The multiple NF-Y subunit combinations result in a large variety of NF-Y complexes and diverse functions (Zhao et al., 2016).

Large body of investigations reveal that NF-Y regulate plant growth, development and environmental stress responses (Hou et al., 2014; Zhao et al., 2016; Swain et al., 2017; Zanetti et al., 2017; Lian et al., 2018; Wang et al., 2018). *Arabidopsis* NF-YC3/4/9 are bound to CCAAT elements of *ABI5* promoter to activate its expression and integrated GA and ABA signaling pathways in seed germination (Liu et al., 2016). NF-YA1/2/4, NF-YB1/2/3, and NF-YC1/2/3/4/9 regulate *Arabidopsis* flowering time (Kumimoto et al., 2008, 2010; Hackenberg et al., 2012; Mu et al., 2013; Hou et al., 2014; Xu et al., 2016). *Arabidopsis* NF-YC1 confers cold tolerance (Shi et al., 2014), NF-YB3 and NF-YC10 confer heat responses (Sato et al., 2014), NF-YA5, NF-YB1, and NF-YC2 confer drought tolerance (Nelson et al., 2007; Li et al., 2008; Liu and Howell, 2010), and NF-YA1 confers salt tolerance (Li Y.J. et al., 2013). Overexpressing *NF-YA5* increased drought tolerance, whereas *Arabidopsis* *nf-ya5* mutants were sensitive to drought compared with the wild type (WT) (Li et al., 2008). *OsNF-YA7* expression was up-regulated by drought, and transgenic rice overexpressing *OsNF-YA7* showed enhanced drought tolerance (Lee et al., 2015). *OsNF-YC13* is identified to be associated with salt tolerance from one activation-tagged salt tolerant *indica* rice (Manimaran et al., 2017). Our previous study showed that expression of *CdtNF-YC1*, an ortholog of *OsNF-YC4* or *AtNF-YC2*, is induced by drought and salt stress in hybrid bermudagrass, which depends on ABA; its overexpression results in elevated drought and salt tolerance in transgenic rice with increased ABA sensitivity (Chen et al., 2015). NF-Ys are good candidate genes used for crops improvement of drought and salt tolerance.

Seashore paspalum (*Paspalum vaginatum* O. Swartz, 2n = 2x = 20) is an important warm-season turfgrass species and commonly used on golf course, athletic fields, and landscape areas. Compared to other turfgrass species, seashore paspalum has superior salt tolerance (Shahba et al., 2012; Uddin et al., 2012), which is associated with maintenance of ion homeostasis by maintaining a high K^+^ concentration in shoots, reducing the Na^+^ being transferred from roots to shoots and leading to maintenance of high K^+^/Na^+^ ratio under salt conditions (Uddin et al., 2012; Guo et al., 2016). However, it is sensitive to drought (Schiavon et al., 2012; Zhou et al., 2012). There is increasing demand for turfgrass species with improved drought and salt tolerance due to limited water resources and increasing salinity soil worldwide and irrigation using saline water (Pessarakli, 2018). Transgenics has provided an effective tool for turfgrass improvement (Guo et al., 2003; Giri and Praveena, 2015; Luo et al., 2017). For example, broad abiotic stress tolerance is enhanced by overexpressing rice SUMO E3 ligase (OsSIZ1) or a cyanobacterial flavodoxin in transgenic creeping bentgrass (*Agrostis palustris* L.) (Li Z. et al., 2013; Li et al., 2017). However, transgenic seashore paspalum with improved abiotic stress tolerance has not been reported. It was aimed of this study to generated transgenic seashore paspalum for improved drought and salt tolerance by overexpressing *CdtNF-YC1*.

## Materials and Methods

### Generation of Transgenic Seashore Paspalum

The sterilized seeds of the seashore paspalum cultivar ‘Sea Spray’ were placed on callus induction medium (MS2.5) (MS basal medium, 2.5 mg L^−1^ 2, 4-dichlorophenoxy acetic acid (2, 4-D), 0.6 mg L^−1^ CuSO_4_, 30 g L^−1^ sucrose, 8 g L^−1^ agar, pH 5.8) and incubated at 25°C for induction of callus in the dark. The compact embryogenic callus was propagated for 3 to 5 months with subcultures every 4 weeks, followed by infection with *Agrobacterium tumefaciens* strain EHA105 (OD_600__nm_ = 0.6) harboring *CdtNF-YC1* construct (Chen et al., 2015) for 30 min at room temperature, with occationally shaking. The infected calli were co-cultivated on solid MS2.5 for 2 days in the dark at 25°C. After co-cultivation, the calli were rinsed two to three times in sterilized water containing 200 mg L^−1^ cefotaxime, drained on filter paper and transferred to callus selection medium (MS2.5 supplemented with 200 mg L^−1^ hygromycin B and 200 mg L^−1^ cefotaxime). After 10 to 12 weeks of selection with subcultures every 4 weeks at 28°C, hygromycin B resistant calli were transferred to regeneration medium with selection pressure (MS basal medium containing 0.2 mg L^−1^ kinetin, 100 mg L^−1^ hygromycin B, and 100 mg L^−1^ cefotaxime) for regeneration at 30°C under light. Regenerated shoots were transferred to 1/2 MS containing 100 mg L^−1^ hygromycin B and 100 mg L^−1^ cefotaxime for developing roots.

### Plant Growth and Treatments

Transgenic plants and WT were planted in soil with a mixture of peat and perlite (3:1) in plastic pots and grown in a greenhouse under nature light. For gene transcription analysis, the third leaves from top were detached and incubated in 0.4 M NaCl solutions for 5 h as salt treatment, or placed in a laminar flow hood for drying gradually as dehydration treatment. The leaf samples were then frozen in liquid nitrogen and used for extraction of total RNA. For physiological measurements, similar size of sods were transplanted into 4 × 8 holes plug tray with the same amount of soil in each hole for growing 2 months. Plants were fully irrigated, followed by withholding irrigation until WT plants showed serious wilting as drought treatment. The leaves were harvested for measurements of relative water content (RWC) and ion leakage, followed by re-irrigating the plants for 7 days of recovery. For salt treatment, plants were irrigated with 0.2 M NaCl solution for 7 days and then 0.4 M NaCl for 7 days, followed by 0.7 M NaCl for 4 days. After the leaves were harvested for measurements of *F*_v_/*F*_m_, chlorophyll, Na^+^ and K^+^ concentrations, plants were re-irrigated for 7 days of recovery.

### Analysis of PCR and Southern Blot Hybridization

Genomic DNA was extracted using the hexadecyltrimethylammonium bromide (CTAB) method. PCR was conducted for detection of *HPT* gene, using forward primer HPT-F (5′-ATGTTGGCGACCTCGTATT-3′) and reverse primer HPT-R (5′-CGTTATGTTTATCGGCACTTT-3′). Southern blot was performed as described previously (Luo et al., 2017). Digoxigenin (DIG) labeled *hpt* was used as DNA probes for hybridization. Probe labeling and hybridization were conducted using DIG High Prime DNA Labeling and Detection Starter Kit II (Roche Diagnostics, Basel, Switzerland) according to the manufacturer’s protocol. Hybridization signals were detected using a chemiluminescence imaging system FUSION SOLO 3S (VILBER, Paris, France).

### Quantitative RT-PCR (qRT-PCR)

Total RNA was extracted by using TRIzol reagent (Life Technologies, Carlsbad, CA, United States) according to the manufacturer’s instructions. First-strand cDNA was synthesized from 1 μg of total RNA using the PrimeScript RT reagent Kit with gDNA Eraser (Takara Bio, Inc., Otsu, Shiga, Japan). A diluted cDNA was used as template for qRT-PCR analysis in a total of 10 μl SYBR Green mix (Takara Bio, Inc., Otsu, Shiga, Japan), and *ACTIN1* was amplified as an internal control to normalize the amount of template. The PCR primers were listed in **Table 1**. Melting profiles which showed a single product specific melting temperature was used to validate the primer specificity. Efficiency of all PCR primers was above 95%.

**Table 1:** Primers used for qRT-PCR.

Gene name	Primer	Sequence (5′–3′)
*CdtNF-YC*	Forward	AAGATTATGAAGGCTGATGAG
	Reverse	TCCACCAAGAAGTCGTAA
*PvACYIN1*	Forward	CTTCTCTCAGCACTTTCCAACA
	Reverse	AAACATAACCTGCAATCTCTCC
*PvLEA3*	Forward	GGGAGAAGGCGATGAACAC
	Reverse	GTGGTGCCGTCCTTGTT
*PvP5CS1*	Forward	CAAGGACGTCAAGCGGATTA
	Reverse	ACCTGCTCACACAAGGAAC
*PvABI2*	Forward	GCCACCTCAGCTTGCTATAA
	Reverse	CTTGGGCATTGAGCCATATTC
*PvDREB1B*	Forward	TCGCTGGCGAAGGAAAC
	Reverse	CATGCCACCGAACCTGAA

### Evaluation of Drought Tolerance

Relative water content and ion leakage and were determined as previously described (Liu et al., 2017). Fresh leaves were weighed (W_f_), followed by immersing in distilled water overnight to obtain the weight of water-saturated leaves (W_S_). Then the leaves were dried in an oven at 80°C for 24 h to obtain the dry weight (W_d_). RWC was calculated using the formula RWC = (W_f_ – W_d_)/(W_S_ – W_d_) × 100. Survival rate was measured after the drought-treated plants were re-watering for 7 days of recovery. Survival rate (%) = number of survived plants after re-watering/total number of plants (Liu et al., 2017). For measurement of ion leakage, leaf samples were immersed in 10 ml of deionized water overnight and the conductivity of the solution (*C*_1_) was measured. The leaf samples were then heated in boiling water bath (100°C) for 20 min. The conductivity of the killed tissues (*C*_2_) was measured again after the samples were chilled to room temperature. Ion leakage was calculated as the percentage of *C*_1_ to *C*_2_.

### Assessment of Salt Tolerance

The third leaf from the top was used for measurements of *F*_v_/*F*_m_ and chlorophyll concentration after 4 days of treatment with 0.7 M NaCl. *F*_v_/*F*_m_ was measured using a pulse-modulated fluorometer (Model FMS-2, Hansatech Instruments, Ltd.) according to the manufacturer’s instructions. For measurement of chlorophyll, the leaves (0.1 g) were ground in a mortar using pestle and extracted for 1 h in 10 ml 80% ethanol (v/v). After filtration, the filtrates were diluted and the absorbance at 663 and 645 nm was determined using a spectrophotometer as described previously (Luo et al., 2017).

### Measurements of Na^+^ and K^+^ Contents

Na^+^ and K^+^ were measured as described by Essah et al. (2003). Leaves and roots were harvested after 4 days of salt treatment with 0.7 M NaCl, followed by drying at 80°C for 24 h. The dry samples were powdered in a mortar with a pestle, and a 100 mg sample was extracted in perchloric acid-nitric acid solution (1:2) overnight, and the solutions were analyzed for Na^+^ and K^+^ contents using a flame photometer (Jenway Model PEP7, United States).

### Data Analysis

Data from three replicates were analyzed by using one-way ANOVA. Results are showed as mean ± standard error of biological replications. The means were separated using Duncan’s multiple range test (*P* < 0.05). All statistical analysis was performed by Statistical Package for the Social Sciences (SPSS 17.0).

## Results

### Analysis of Transgenic Plants

**FIGURE 1:**
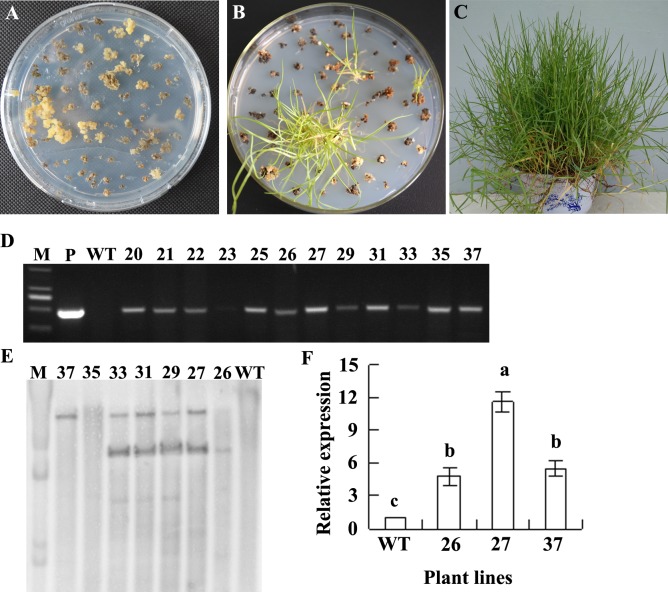
Generation and analysis of transgenic plants. *Agrobacterium*-infected calli were placed on selection medium containing 200 mg L^−1^ hygromycin **(A)**. The hygromycin-resistant calli were exposed to regeneration **(B)**. The regenerated plants were growing in soil **(C)**. *HPT* gene was detected in transgenic plants by using PCR **(D)** and DNA blot hybridization **(E)**. M indicates DNA marker; P indicates plasmid containing *CdtNF-YC* gene; and WT indicates wild type (WT). Relative expression of *CdtNF-YC* was detected using real time quantitative RT-PCR, and *Actin* gene was used as a reference **(F)**. Means of three replicates and standard errors are presented; the same letter above the column indicates no significant difference at *P* < 0.05.

The embryogenic calli of seashore paspalum infected with *Agrobacterium* *tumefaciens* containing the *CdtNF-YC1* were cultured on the selection medium containing hygromycin B (**Figure 1A**), followed by transferring the resistant calli to regeneration medium for regeneration (**Figure 1B**). The putative transgenic plants were planted in plastic pots filled with soil and growing in a greenhouse (**Figure 1C**). After detection of transgene *HPT* using PCR (**Figure 1D**), the PCR positive plants were detected using DNA blot hybridization. One single *HPT* hybridization signal was detected in two transgenic lines (26 and 37) and two similar *HPT* hybridization signals were detected in four lines (27, 29, 31, and 33), while no signal was shown in WT plants (**Figure 1E**), indicating an integration of the transgene into the genomes of transgenic seashore paspalum plants. Relative expression of *CdtNF-YC1* was analyzed in the independent transformant lines (26, 27, and 37) using quantitative RT-PCR (qRT-PCR). Compared to the WT, higher transcript levels of *CdtNF-YC1* were observed in transgenic lines. Line 27 had the greatest *CdtNF-YC1* expression, while the similar transcript level was observed in lines 26 and 37 (**Figure 1F**).

### Transgenic Plants Had Improved Drought Tolerance

**FIGURE 2:**
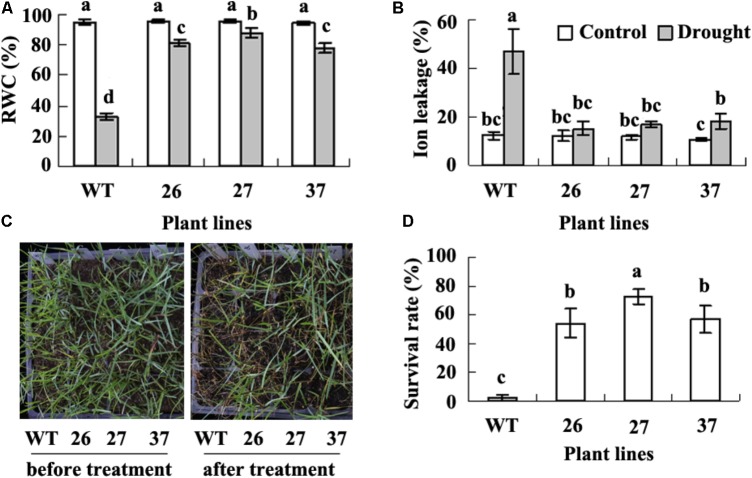
Analysis of drought tolerance in transgenic plants in comparison with the WT. Relative water content (RWC, **A**) and ion leakage **(B)** was measured when WT plants displayed wilting after withholding irrigation. Photography **(C)** was taken before withholding irrigation (left) and 7 days after re-watering wilted plants caused by withholding irrigation (right), followed by recording survival plant numbers to calculate survival rate **(D)**. Means of three replicates and standard errors are presented; the same letter above the column indicates no significant difference at *P* *<* 0.05.

There was no difference in morphologic phenotype between transgenic plants and WT. Previous study showed that *CdtNF-YC1* overexpression results in elevated tolerance to drought and salt in transgenic rice (Chen et al., 2015). To investigate the effect of *CdtNF-YC1* on drought tolerance in seashore paspalum, we measured RWC and ion leakage. A greatly decreased RWC and increased ion leakage were observed in WT, while RWC and ion leakage were not significantly altered in transgenic plants after drought treatment (**Figures 2A,B**). Almost all of WT plants became dead after 20 days of withholding irrigation (1.6% survival rate), while transgenic plants had 54 to 56% survival rate (**Figures 2C,D**). The results indicated that overexpression of *CdtNF-YC1* resulted in an enhanced drought tolerance in transgenic seashore paspalum plants.

### Transgenic Plants Showed an Enhanced Salt Tolerance

**FIGURE 3:**
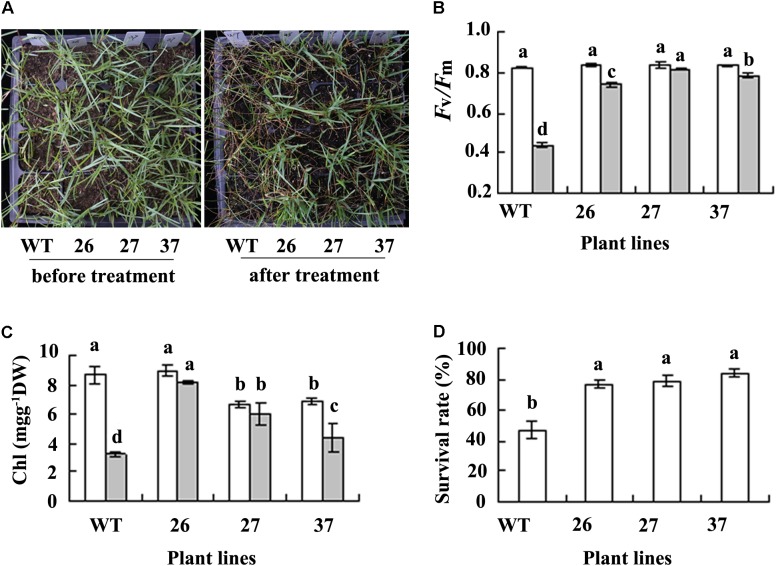
Analysis of salt tolerance in transgenic plants in comparison with the WT. Photography **(A)** was taken before salt treatment (left) and 7 days after watering to dilute slat as a result of treatment with 0.7 M NaCl (right), followed by recording survival plant numbers to calculate survival rate **(D)**. *F*_v_/*F*_m_ **(B)** and chlorophyll (Chl, **C**) were measured 4 days after NaCl concentration was increased to 0.7 M. Means of three replicates and standard errors are presented; the same letter above the column indicates no significant difference at *P* < 0.05.

A serious damage symptom was observed in WT when NaCl concentration was increased to 0.7 M for 4 days, while transgenic plants were little damaged (**Figure 3A**). *F*_v_/*F*_m_ and chlorophyll concentration were decreased greatly in WT plants after salt treatment, while they were unaltered or slightly decreased in transgenic plants (**Figures 3B,C**). WT plants had a survival rate of 47% after salt treatment, while transgenic had survival rate of 77 to 84% 7 days after watering to remove salt from the soil (**Figures 3A,D**). The results indicated that transgenic plants had increased salt tolerance compared with WT plants.

**FIGURE 4:**
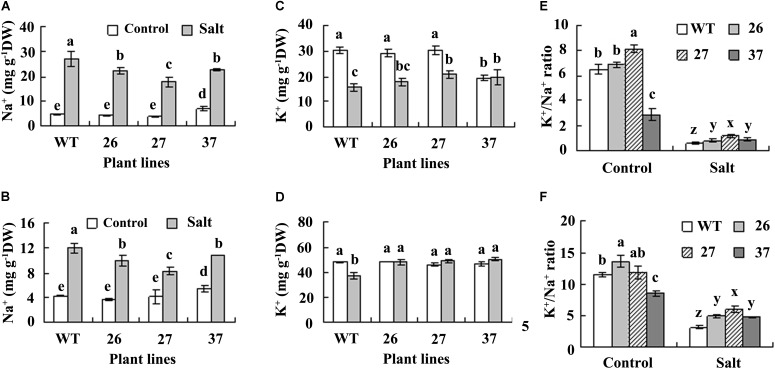
Analysis of Na^+^, K^+^, and K^+^/Na^+^ ratio in roots **(A,C,E)** and leaves **(B,D,F)** of transgenic plants and the WT. Roots and leaves were sampled before treatment (control) and 4 days after NaCl concentration was increased to 0.7 M for measurement of Na^+^ and K^+^. Means of three independent experiments and standard errors are presented; the same letter above the column indicates no significant difference at *P* *<* 0.05.

Maintenance of ion homeostasis is important for plants adaptation to salt environment. Na^+^ and K^+^ contents showed no difference in both roots and leaves between transgenic plants and WT under control condition, except for a higher Na^+^ content in leaves of line 37 (**Figures 4A–D**). Na^+^ content was increased in both roots and leaves in all plants after salt treatment, while lower level was maintained in transgenic plants as compared with WT (**Figures 4A,B**). K^+^ was decreased in roots of both WT and transgenic lines 26 and 27, but unaltered in line 37 after treatment with salt (**Figure 4C**). Salt treatment resulted in decrease in K^+^ level in WT leaves, but did not alter that in transgenic plants (**Figure 4D**). K^+^/Na^+^ ratio was decreased in all plants after salt treatment, and higher K^+^/Na^+^ ratio was maintained in transgenic plants than in WT (**Figures 4E,F**).

### Analysis of Expression of ABA and Stress Responsive Genes

**FIGURE 5:**
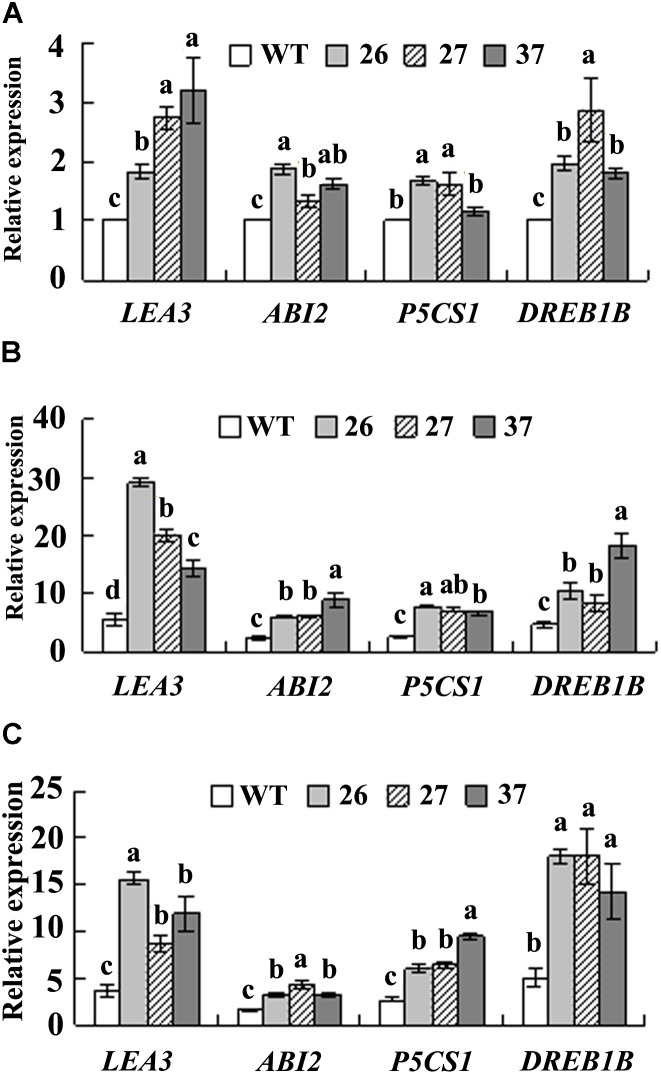
Analysis of transcript levels of *PvLEA3*, *PvABI2*, *PvP5CS1*, and *PvDREB1B* in the transgenic plants in comparison with the WT. Detached leaves were placed in water for 4 h as control **(A)**, or air-dried in a hood for 4 h as dehydration treatment **(B)**, or placed in 0.4 M NaCl solution for 5 h as salt treatment **(C)**. *PvACTIN1* was used as a reference gene for qRT-PCR. Relative expression of each gene was calculated by defining the normalized transcript level in WT plant under control condition as one. Means of three replicates and standard errors are presented; the same letter above the column indicates no significant difference at *P* *<* 0.05.

Three ABA responsive genes, *PvLEA3, PvP5CS1*, and *PvABI2*, and one stress responsive gene *PvDREB1B*, were analyzed under control and stress conditions. Higher transcript levels of *PvLEA3*, *PvABI2*, and *PvDREB1B* were observed in three transgenic lines, and higher *PvP5CS1* transcript levels in lines 26 and 37 as compared with WT under control condition (**Figure 5A**). Dehydration and salt stress resulted in enhanced expression of *PvLEA3, PvP5CS1*, *PvABI2*, and *PvDREB1B* in all plants, while transgenic lines had higher levels than WT (**Figures 5B,C**).

## Discussion

Transgenic approaches provide an effective tool to improve crops traits. Most of the commercial seashore paspalum cultivars are cultivated using sprigs or sods, while ‘Sea Spray’ is the only seeded cultivar. *Agrobacterium*-mediated transformation of seashore paspalum has been reported using an unnamed cultivar (Kim et al., 2009). In this study, transgenic seashore paspalum overexpressing *CdtNF-YC1* were generated and identified. The transgene was detected in the genomes of transgenic lines based on Southern blot analysis. qRT-PCR assay indicated that *CdtNF-YC1* gene was expressed in transgenic lines. NF-Ys are involved in plant adaptation to environmental stresses (Swain et al., 2017). Except for those from model plants, several NF-Y genes have been identified to confer abiotic stress tolerance. For example, overexpression of a drought responsive *ZmNF-YB16* improves drought resistance and yield inducing antioxidant protection and enhancing photosynthesis in transgenic maize (Wang et al., 2018). *CdtNF-YC1* has been documented to increase drought and salt tolerance in transgenic rice (Chen et al., 2015). Likely, overexpressing *CdtNF-YC1* led to increased drought and salt tolerance in transgenic seashore paspalum in this study. This is the first report on transgenic seashore paspalum with modified traits.

Expression of *LEA3, P5CS1*, *ABI2*, and *DREB1B* genes is up-regulated by *CdtNF-YC1* in transgenic rice, and CCAAT *cis*-acting elements exist in the promoter regions of these genes (Chen et al., 2015). Likely, transcript levels of *PvLEA3, PvP5CS1*, *PvABI2*, and *PvDREB1B* were induced by drought and salt stress, and higher levels were observed in transgenic plants compared with WT, although the existence of CCAAT *cis*-acting elements in the promoter regions of these genes in seashore paspalum was not analyzed due to the absence of genomic information. Up-regulation of these genes leads to increased drought and/or salinity tolerance (Xu et al., 1996; Ohta et al., 2003; Datta et al., 2012; Per et al., 2017). Plant molecular responses to abiotic stress include ABA-dependent and -independent pathways. *LEA3, P5CS1*, and *ABI2* are ABA-dependent stress responsive marker genes (Yamaguchi-Shinozaki and Shinozaki, 2006). NF-Ys have been validated to be involved in ABA signaling in plant responses to drought (Li et al., 2008; Chen et al., 2015; Bi et al., 2017). *DREB1B* is responsive to stress and functions in ABA-independent pathway (Zhu, 2002), which was induced in *CdtNF-YC1* transgenic rice (Chen et al., 2015) and seashore paspalum (this study). It is suggested that the enhanced drought and salt tolerance in transgenic seashore paspalum is associated with up-regulation of a series of stress responsive genes in both ABA-dependent and ABA-independent pathway by *CdtNF-YC1*.

Maintenance of ion homeostasis is important for plant adaptation to saline environment. Plants may increase salt tolerance either by minimizing the entry of salt into plants or by ion compartmentation mechanism to minimize the salt concentration in the cytoplasm. Plants may restrict uptake of ions like Na^+^ and Cl^−^ or selectively take up ions to maintain a higher cellular K^+^/Na^+^ ratio in saline environments (Munns and Tester, 2008). Among six warm-season turfgrass species, seashore paspalum had the highest salt tolerance with lowest accumulation of Na^+^, least reduction of K^+^ and highest K^+^/Na^+^ ratio after salt stress (Uddin et al., 2012). In this study, salt treatment resulted in increased Na^+^ level and decreased K^+^ level in seashore paspalum plants, while lower levels of Na^+^ and altered K^+^ level were maintained in transgenic plants, which led to a higher K^+^/ Na^+^ ratio in roots and leaves in transgenic plants than in WT. Many genes such as those encoding Na^+^/H^+^ antiporters, NHX antiporters and HKT transporters are involved in homeostasis of K^+^ and Na^+^ in plant cells in response to salt stress (Munns and Tester, 2008; Almeida et al., 2017). Although the ion homeostasis related genes regulated by CdtNF-YC1 were not revealed in the present study, our results suggest that overexpression of *CdtNF-YC1* influences maintenance of ion homeostasis under salt stress conditions. It will be interesting to investigate the regulation of PvNF-Ys on maintenance of ion homeostasis in response to salt stress for understanding salt tolerance mechanism in seashore paspalum in the future.

In summary, transgenic seashore paspalum plants overexpression *CdtNY-YC1* with enhanced drought and salt tolerance were obtained in the present study. The enhanced drought and salt tolerance is associated with induction of a series of stress responsive genes by *CdtNF-YC1* in both ABA-dependent and ABA-independent pathway. In addition, the improved salt tolerance was also associated with maintenance of Na^+^ and K^+^ homeostasis under salt stress in transgenic plants.

## Author Contributions

XW conducted the experiments and wrote the manuscript. HS and ZG designed the experiments and revised the manuscript.

## Conflict of Interest Statement

The authors declare that the research was conducted in the absence of any commercial or financial relationships that could be construed as a potential conflict of interest.
